# Cognitive Reserve in Model Systems for Mechanistic Discovery: The Importance of Longitudinal Studies

**DOI:** 10.3389/fnagi.2020.607685

**Published:** 2021-01-21

**Authors:** Joseph A. McQuail, Amy R. Dunn, Yaakov Stern, Carol A. Barnes, Gerd Kempermann, Peter R. Rapp, Catherine C. Kaczorowski, Thomas C. Foster

**Affiliations:** ^1^Department of Pharmacology, Physiology and Neuroscience, University of South Carolina School of Medicine, Columbia, SC, United States; ^2^The Jackson Laboratory, Bar Harbor, ME, United States; ^3^Cognitive Neuroscience Division, Department of Neurology, Vagelos College of Physicians and Surgeons, Columbia University, New York, NY, United States; ^4^Departments of Psychology and Neuroscience, University of Arizona, Tucson, AZ, United States; ^5^Evelyn F. McKnight Brain Institute, University of Arizona, Tucson, AZ, United States; ^6^CRTD—Center for Regenerative Therapies Dresden, Technische Universität Dresden, Dresden, Germany; ^7^German Center for Neurodegenerative Diseases (DZNE), Helmholtz Association of German Research Centers (HZ), Dresden, Germany; ^8^Laboratory of Behavioral Neuroscience, Neurocognitive Aging Section, National Institute on Aging, Baltimore, MD, United States; ^9^Department of Neuroscience, McKnight Brain Institute, University of Florida, Gainesville, FL, United States; ^10^Genetics and Genomics Program, University of Florida, Gainesville, FL, United States

**Keywords:** longitudinal studies, animal models, cognitive reserve, brain maintenance, biological markers

## Abstract

The goal of this review article is to provide a resource for longitudinal studies, using animal models, directed at understanding and modifying the relationship between cognition and brain structure and function throughout life. We propose that forthcoming longitudinal studies will build upon a wealth of knowledge gleaned from prior cross-sectional designs to identify early predictors of variability in cognitive function during aging, and characterize fundamental neurobiological mechanisms that underlie the vulnerability to, and the trajectory of, cognitive decline. Finally, we present examples of biological measures that may differentiate mechanisms of the cognitive reserve at the molecular, cellular, and network level.

## Introduction

Differences in the onset and trajectory of age-related cognitive decline result from factors related to the characteristics of the individual (genetics, sex, and epigenetic dispositions), the environment, and lifestyle factors, as well as positive experiences (e.g., environmental enrichment) and negative experiences (e.g., stress or inflammation), which interact to influence the structure and function of molecules, cells, and circuits that comprise the brain. An understanding of how these factors interact across the lifespan and propagate through multiple scales of biology will enable interventions that can maintain the brain and promote the formation of “reserve,” a term that describes plastic properties of the brain that collectively allow for sustained cognitive performance in the face of age-related changes, brain insult, or disease. An international consortium of researchers across a wide range of disciplines, covering both human and nonhuman animal studies, currently works on consensus definitions for the related concepts “cognitive reserve,” “brain reserve,” “brain maintenance” as well as “resilience” and “resistance”^1^. A “white paper” and several other publications have previously started first attempts to harmonize nomenclature and concepts (Cabeza et al., 2018; Stern et al., 2020). Within these efforts, it was recognized that longitudinal studies are a central requirement to examine variability in the trajectory of cognitive decline. The trajectory of age-related changes in cognition and measures of brain aging is not necessarily linear. Measurements at multiple time points permit the characterization and analysis of how chronological age interacts with nonlinear trajectories of aging. The goal of the present article is to more precisely identify and characterize the role of longitudinal animal studies in this context.

Human research, aimed at further elucidating cognitive reserve, requires the inclusion of three components: clinical or cognitive performance changes and outcomes, the status of brain aging, including metrics such as gray matter volume, cortical thickness, white matter tract integrity, or white matter hyperintensity burden (reflecting age-related brain change or pathology), and a measure of the reserve itself—“reserve” here used as an umbrella term for the mentioned inter-related concepts. Similarly, nonhuman animal studies (from here forward referred to as animal studies) can examine cognitive performance changes and outcomes for a variety of domains of cognition of which the neural underpinnings are often well understood. Moreover, these tests can be done under highly controlled conditions and can be repeatedly applied over the lifespan. Brain imaging has been employed in animal models to examine the status of brain aging, providing the opportunity to translate findings across species. An additional advantage of animal models is the ability to probe levels of biology in-depth at the cellular and molecular levels that cannot be examined in humans. In this case, the relationship of cognition and molecular or cellular measures may help define concepts related to cognitive reserve, including brain maintenance (i.e., the similarity in biological measures between young and aged-unimpaired). Whereas, for cognitive reserve, measured as a change in a process (resilience, adaptation, or compensation) in response to brain aging, better-than-expected cognition may result in biological measures quite different from that normally observed in young adults or aged-impaired animals.

Within the human literature, the terms brain maintenance and cognitive reserve are generally used to define better than expected cognition for a given age or in the face of brain pathology. In the case of brain maintenance, preserved cognition is due to delaying brain changes, structural, physiological, molecular, or cellular, associated with aging, to maintain youthful brain structure and function. In contrast, cognitive reserve is thought to mediate better cognition for a given level of brain aging or pathology. The mechanisms of the cognitive reserve are not clear and so cognitive reserve proxies are employed. Typical proxies for the degree of cognitive reserve include IQ, cognitively stimulating exposures across the age span, education, occupational attainment, leisure activity, social networks, or other exposures that might impart reserve (Oveisgharan et al., 2020). Similar proxies (baseline cognition, activity levels, social isolation, and environmental enrichment) may be established for longitudinal animal studies. Alternatively, animal studies might focus on inherent genetic or epigenetic modifications to understand individual differences in cognition. A key aspect of such studies must always be the variability of these parameters within a given human or animal population and the impact of aging on this variability. Reserve is a highly individual measure. Animal studies allow one to control for genetic (and to some extent environmental) contributions not only to the expression of a phenotype but also its variance (Dunn et al., 2020). Studies that address a larger number of variables are thus at an advantage because it allows the description of complex matrices of covariance. Whereas in the human situation, the impact of unknown parameters is difficult to estimate, well-designed animal studies enable model building that helps estimate such contributions (and their individual variability).

Finally, animal studies permit manipulations of molecules and circuits to test hypothesized mechanisms. Furthermore, longitudinal animal studies permit examination of well-controlled environments or environmental experimental modifiers that may positively (exercise, Mediterranean diet, environmental enrichment) or negatively (inflammation, cardiovascular disease, Western diet, stress) impact the propensity for cognitive decline (Dunn et al., 2019; Neuner et al., 2019b). Such studies can illuminate the timing, critical periods, and genetic context thought to influence the trajectory of cognitive decline.

The goal of this review article is to provide a resource for longitudinal studies, using animal models, directed at understanding the relationship between cognition and brain structure and function throughout life and the mechanisms of maintenance and reserve that modify this relationship to support successful cognitive function in aging. We propose that longitudinal studies will build upon knowledge gained using a prior cross-sectional design to identify early predictors of variability in cognitive function during aging and characterize fundamental neurobiological mechanisms that underlie the vulnerability to and the trajectory of cognitive decline. Finally, we present examples, mainly from cross-sectional studies, of biological measures that may differentiate mechanisms of the cognitive reserve at the molecular, cellular, and network level.

## Advantages and Limitations of Longitudinal Studies in Animal Models

Compared to cross-sectional studies, there are both advantages and limitations of conducting longitudinal studies in animal models to examine individual differences in cognitive aging trajectories. In the case of rodent models, an advantage is that there is considerable prior knowledge about cognitive aging. Additionally, the shorter lifespan, and ability to modulate the extent of genetic diversity, as well as the ability to control and exploit environmental factors, facilitates examination of interventions (Burke and Foster, 2019; Dunn et al., 2019). Nevertheless, a challenge for animal studies modeling cognitive decline over the lifespan is to identify age-sensitive tasks that can be used to translate findings across different levels of analysis and different species to effectively characterize the trajectory of cognitive decline (Foster, 2012a). Relative to cross-sectional research, longitudinal studies can better identify the onset of cognitive impairment and provide a potential time course for changes in theoretical constructs of cognition and health, such as episodic memory, physical function, and circadian dysregulation. Also, longitudinal testing can control for batch effects and guard against problems associated with a history of exposure to an impoverished environment (Sabolek et al., 2004; Volkers and Scherder, 2011). For example, in cross-sectional studies, cognitive impairment and increased anxiety of the oldest animals arise due to an interaction of age and the length of exposure to social isolation or an impoverished environment (Diamond, 1990; Winocur, 1998; Bell et al., 2009; Diniz et al., 2010; Volkers and Scherder, 2011; Sampedro-Piquero et al., 2014; Sparling et al., 2018; Wang et al., 2018). In contrast, animals that are repeatedly handled and tested across the lifespan exhibit reduced anxiety to novel situations (Dellu et al., 1994; Hall et al., 1997; Febo et al., 2020). In this case, repeated testing may be considered a form of environmental enrichment, which reduces anxiety. Environmental enrichment itself is a key experimental concept in this context as it has allowed the dissection of non-genetic effects on the development of brain-related phenotypes, including susceptibility to disease (Nithianantharajah and Hannan, 2006; Kempermann, 2019). Exposure to environmental enrichment can, among other effects, reduce anxiety and improve cognition (Leal-Galicia et al., 2008; Hughes and Collins, 2010; Kumar et al., 2012; Garthe et al., 2016; Cortese et al., 2018; Sparling et al., 2018; Birch and Kelly, 2019). In addition to unintended (or at least unidentified) enrichment effects, longitudinal studies of cognition need to control for influences that carry over from one testing situation to another, including memory for procedural aspects of the behavioral tasks (Guidi et al., 2014).

A key advantage of animal models is the ability to experimentally examine and manipulate cellular, molecular, and epigenetic mechanisms that accompany changes in cognitive function, which may suggest possible similar roles in humans. However, success in translating findings across different levels of analysis and different species will depend on having age-sensitive tasks, linked to defined neural systems (Foster, 2012a; Roberson et al., 2012). Furthermore, to examine individual variability in the trajectory of cognitive aging will require tasks that can be repeated throughout the full lifespan or the use of different procedures that independently assess and interrogate the same cognitive process or integrity of the same neural system. To achieve such analogous testing across species boundaries is no trivial task. Touchscreen tasks and virtual humanized versions of the classical Morris watermaze task for hippocampal learning are an example of such efforts (Foster et al., 2012; Horner et al., 2013; Dickson et al., 2014; Beraldo et al., 2019). A touchscreen-based attentional set-shifting task, modeled after the CANTAB intra-extra dimensional set-shifting task in human studies, measures discrimination of simple and multidimensional visual stimuli to reveal individual differences in attentional set-shifting deficits from simple visual discrimination—and expected to control for age-related vision changes that can confound interpretation of deficits in executive function. During testing on this operant task, mice learn to discriminate pairs of visual stimuli of a single dimension presented on the touch screen in response to reward (vanilla soymilk). Once the criterion is reached, correct performance on discrimination of compound stimuli from novel exemplars is assessed, rewarded, and thus evaluates intra-dimensional shift reversal learning. The extra-dimensional shift requires attention to previously unrewarded dimensions of the compound stimuli where performance is defined by errors to criterion, latency to make a choice, latency to collect the reward, and propensity to correct reward. Given the applicability of this task for longitudinal measures of visual discrimination, reversal learning, and attentional set-shifting deficits in aging mice, future efforts to identify and fully characterize cellular, molecular, and epigenetic mechanisms that accompany such changes in cognitive function may be realized (Dickson et al., 2014). The Morris watermaze test of episodic spatial memory developed in rodents has been adapted using virtual computer-generated environments for testing of spatial memory in humans with controls that can compensate for age-related changes in reaction time and mobility—bridging methodologies for studies of cognitive aging in rodent and human behavioral studies (Foster et al., 2012).

Similarly, the use of analogous techniques in humans and animals, such as brain imaging, could be used to identify the time course for neural network alterations. At the same time, the opportunity to validate imaging results histologically, at least at defined study endpoints, provides a fundamental advantage of animal studies. In turn, correlations between behavior and brain measures could illuminate their relationship to cognitive/brain reserve, suggesting when and where to examine cellular and molecular changes, and address questions of when to successfully apply experimental manipulations designed to tap into mechanisms of cognitive/brain reserve in humans and animal models (Roberson et al., 2012).

At present, the experimental literature using longitudinal studies in animals is small in comparison to the body of cross-sectional data. Many cross-sectional studies imply interactions between trajectories of age-related cognitive decline and advancing age, but only a comparison between effects at different age points has been performed. Only a within-animal design, however, permits the characterization of individual differences in the trajectory of cognitive decline. It is thus important to invest in more longitudinal studies and to promote the methodology, analyses, and theoretical background of this approach, and to either validate or invalidate conclusions made from findings derived from cross-sectional approaches.

As the first step in this review of the literature, we identify some relevant variables (age, sex, and strain) and appropriate dependent variables emphasizing psychological measures and cognitive function that can be repeatedly tested. The literature review focuses on behavioral/cognitive measures, linked to specific brain systems that decline with aging in humans and animal models including hippocampal involvement in memory and pattern separation, prefrontal processes of sustained attention, cognitive flexibility and impulsivity, and the perirhinal cortex in recognition memory (Table 1). We highlight considerations for translating findings across different levels of analysis to predict the onset and trajectory of cognitive impairment. Therefore, whenever possible, previous longitudinal studies are underscored to identify possible confounds and recommend best practices (Table 1).

**Table 1:** Longitudinal testing of traits relevant to aging, cognition.

Domain	Assay	Specific trait measured (if applicable)	Effect of age	Considerations
**Physical**
	Bodyweight		↑ With age (Turturro et al., 1999; Altun et al., 2007; Freund et al., 2013; Febo et al., 2020).	Bodyweight may impact motor behavior. Body composition is strain-dependent.
	Grip strength		↓ With age (Fischer et al., 2015; Zhang et al., 2015; Xue et al., 2016; Martinez de Toda et al., 2018; Sheth et al., 2018; Zammit et al., 2019; Febo et al., 2020).	Grip strength predicts cognitive decline in humans butt not rodents (Praetorius Bjork et al., 2016; Stijntjes et al., 2017; McGrath et al., 2019; O’Connell et al., 2019; Febo et al., 2020).
	Gait, rotarod	Motor coordination	↓ With age (Zhang et al., 2015; Bair et al., 2019).	Unclear if related to cognitive function. Cognitive function is impaired when paired with physical demands in the task (Seidler et al., 2010).
	Locomotor activity		↓ With age (Salvatore et al., 2009; Stowie and Glass, 2015; Febo et al., 2020).	Not subject to test/retest or practice effects (Altun et al., 2007). Reduced physical activity corresponds to reduced cognitive function (Shoji et al., 2016; Logan et al., 2018; Febo et al., 2020). Female rodents have higher physical activity compared to males; may be mediated by estradiol and contribute to b, better cognitive performance (Stern and Murphy, 1972; Gerall et al., 1973; Gentry and Wade, 1976; Yonker et al., 2006; Bean et al., 2015; Stowie and Glass, 2015; Barha et al., 2017; Rosenfeld, 2017; Febo et al., 2020). Voluntary locomotor activity increases neurogenesis and synaptic plasticity (Kronenberg et al., 2006).
	Visual, hearing acuity		↓ With age (strain-dependent)	Age-related memory decline is not due to loss of visual acuity (Lindner and Gribkoff, 1991; Weber et al., 2015).
	Sleep		↑ Fragmentation with age (Zepelin et al., 1972; Rosenberg et al., 1979; Van Gool and Mirmiran, 1983).	Sensitive to and interacts with age, sex, stress, environment (Mirmiran et al., 1982; Buechel et al., 2014; Fischer et al., 2015). Fragmented sleep is associated with impaired learning (Markowska et al., 1989; Stone et al., 1989).
**Psychological**
	Open field test	Anxiety	↓A novelty-induced locomotor activity with age ↓ or ↑ anxiety with age, depending on the testing paradigm	Reduced center time is associated with decreased learning abilities when cross-sectionally measured (Dellu et al., 1994) or predicts better performance when longitudinally tested (Febo et al., 2020). Neophobia increases with age, extended impoverished environment (Diamond, 1990; Hall et al., 1997; Winocur, 1998; Hellemans et al., 2004; Bell et al., 2009; Diniz et al., 2010; Volkers and Scherder, 2011; Sampedro-Piquero et al., 2014; Sparling et al., 2018; Wang et al., 2018).
				Longitudinal: reduced locomotor activity in OFT with repeated handling, environmental enrichment (Fox et al., 2006; Galani et al., 2007; Leal-Galicia et al., 2008; Hughes and Collins, 2010; Sampedro-Piquero et al., 2014). Cross-sectional: variable effects of age on novelty-induced locomotor activity in OFT (Miyagawa et al., 1998; Boguszewski and Zagrodzka, 2002; Torras-Garcia et al., 2005; Bergado et al., 2011; Meyza et al., 2011; Moretti et al., 2011; Shoji and Miyakawa, 2019).
	Watermaze (visual platform)	Anxiety, spatial learning	Dependent on anxiety response	Repeated training with visual platform elicits reduced anxiety and reduces age-related differences (Rapp et al., 1987; Treit and Fundytus, 1988; Herrero et al., 2006; Guidi et al., 2015).
**Cognitive—hippocampal**
	T-maze	Spatial working memory, reference memory	↓ Working memory, reversal with age (Ando and Ohashi, 1991; Dellu et al., 1997; Zhuo et al., 2007; Talboom et al., 2014; Birch and Kelly, 2019). ↑ Reference memory with age (Talboom et al., 2014). ↑ Acquisition with age, practice (Dellu et al., 1994).	Severe food deprivation improves function in old rats (Ando and Ohashi, 1991). Environmental enrichment, practice protects against age-related decline, with possible female sex-specificity (Dellu et al., 1997; Zhuo et al., 2007; Talboom et al., 2014; Birch and Kelly, 2019).
	Radial arm maze	Spatial working memory	↓ With age (Dellu et al., 1997; Templer et al., 2019).	Continuous training may mask age-related decline (Beatty et al., 1985; Bierley et al., 1986; Caprioli et al., 1991). Increased number of arms, reduced training prevents the use of response strategies in aged animals (Chrobak et al., 1995; Dellu et al., 1997; Sabolek et al., 2004; Templer et al., 2019).
	Y-maze	Spatial, episodic working memory	↔ Spontaneous alternations with age (Chaney et al., 2018; Neuner et al., 2019b). ↓ Performance in delayed choice paradigm with age (Dellu et al., 1994; Vallee et al., 1999).	Variability in performance at midlife may predict future deficits (Stone et al., 1997). Strain differences in mouse performance (Neuner et al., 2019b). Performance may be influenced by novelty-induced locomotor activity (Dellu et al., 1994).
	Watermaze	Working memory, reference memory, episodic memory	↓ Acquisition, delayed matching-to-place (Lindner and Gribkoff, 1991; Clark et al., 1992; Rick et al., 1996; Gallagher et al., 2015; Febo et al., 2020). ↔ or ↓ Reference memory with age (Colombo and Gallagher, 2002; Zhuo et al., 2007).	Early or repeated training is protective against age-related decline in normal aging (but not in Alzheimer’s models; Algeri et al., 1991; Pitsikas et al., 1991; Gyger et al., 1992; Dellu et al., 1997; Vallee et al., 1999; Markowska and Savonenko, 2002; van Groen et al., 2002; Vicens et al., 2002; Billings et al., 2005; Reichel et al., 2017). Correlates to open field performance (Febo et al., 2020). Sensitive to stress effects (Rapp et al., 1987; Treit and Fundytus, 1988; Herrero et al., 2006; Guidi et al., 2015).
	Barnes maze	Spatial working memory		Sensitive to training or practice effects (Heimer-McGinn et al., 2020).
				Less physically demanding, stress-inducing test may be advantageous over watermaze in aged rodents (Harrison et al., 2009; Berdugo-Vega et al., 2020).
	Pattern separation		↓ With age (Yassa and Stark, 2011; Holden and Gilbert, 2012; Gracian et al., 2013; Johnson et al., 2017; Ces et al., 2018; Smith et al., 2020).	Age-related deficits may be related to altered synaptic plasticity and neurogenesis in the dentate gyrus (Wilson et al., 2006; Creer et al., 2010; Wu et al., 2015; Ces et al., 2018; Ray et al., 2018; Smith et al., 2020).
**Cognitive-prefrontal cortex**	5-choice serial reaction time task	Sustained attention	↓ with age (Muir et al., 1999).	Shorter cue duration reveals greater age-related changes (Muir et al., 1999).
	Attention set-shifting task	Cognitive flexibility	↓ With age (Zhuo et al., 2007)	
	Delayed non-matching to position task		↔ With age (longitudinal testing between 24 and 36 months; Blokland et al., 2004).	
	Span task		Variable (Sabolek et al., 2004).	Possibly subject to practice effects.
	Delay discounting	Impulsivity	↓ With age (Simon et al., 2010; Hernandez et al., 2017).	No existing longitudinal testing data. Significant variability across strains exists in the ability to tolerate a delay (Isles et al., 2004; Anderson and Woolverton, 2005; Wilhelm and Mitchell, 2009). Delay tolerance may be associated with working memory, cognitive flexibility (Hernandez et al., 2017).
**Cognitive-perirhinal cortex**	Novel object recognition		↓ With age (Chaney et al., 2018).	Environmental enrichment, previous training protects against age-related decline (Birch and Kelly, 2019).

*↑ = increased with age; ↔ = no change with age; ↓ = decreased with age*.

## Sex Differences

In humans, the pathological progression of age-related neurodegenerative diseases is sexually dimorphic and longitudinal studies in mice appear to confirm sex differences (Havas et al., 2011; Wood et al., 2011; van Duijn et al., 2013; Roy et al., 2018). In most cases, sex differences that have been found in rodent models cannot be easily extrapolated to the human situation, either concerning effect size or underlying genetic cause. The detection of sex differences, however, will be an important co-variate in complex models aimed at elucidating other important relationships (Jonasson, 2005; Sutcliffe et al., 2007; Barter et al., 2019; Febo et al., 2020). Although there are likely interactions of age × genetics × sex (Dunn et al., 2019; Neuner et al., 2019b; O’Connell et al., 2019), cross-sectional studies have reported sex differences in aging of physical and cognitive function (Andrews, 1996; Veng et al., 2003; Jonasson, 2005; Barha et al., 2017; Berkowitz et al., 2018). Also, a few longitudinal studies have confirmed sex differences in rats (Altun et al., 2007; Talboom et al., 2014; Febo et al., 2020).

## Model Differences

The choice of animal model will depend on the questions to be addressed. Due to the ability to modify genetics, mice are generally employed for examining genes linked to aging and diseases of aging (Yuan et al., 2011; Ackert-Bicknell et al., 2015). The relatively short lifespan of rodents allows studies that can address cognitive health span (the period of life spent in good health, unaffected by age-related diseases or disabilities) vs. the entire life span (e.g., Leduc et al., 2010).

The inbred C57BL/6J mouse, and the related C57BL/6JNia substrain supported by the National Institute of Aging for studies of aging, are the most widely used mouse strains for aging research, including genetic manipulations (Mitchell et al., 2015). However, some aging studies have used other strains due to age-related hearing loss in C57BL/6J and related substrains (Johnson et al., 1997; Tremblay et al., 2012).

The availability of murine genetic reference populations, for example, the panel of recombinant inbred strains of mice such as BXD, based on a cross between C57BL/6J and DBA/2J, allows powerful genetic studies of aging that can be related to human cohort studies (Hook et al., 2018). Based on this genetic approach, disease models have been developed, for example, the ongoing AD-BXD study (Neuner et al., 2019b), which represents the first study addressing the interaction between a disease-causing mutation for Alzheimer’s disease and systematic variation of the genetic background.

For aging studies in rats, the inbred Fischer 344 are the most widely used, again due to support by the National Institute of Aging for this model. In the absence of a *de novo* mutation, inbred animals are genetically identical. The similarity in genes should reduce phenotypic variability, increasing reproducibility and fewer animals are required to determine experimental outcomes (Festing, 1976). In turn, the reduced variability is thought to lead to better predictability. This may be a consideration for studies examining independent variables hypothesized to modify cognitive/brain reserve, because any differences observed are likely to be due to treatment effects. Alternatively, there are also outbred rat strains—such as the Sprague–Dawley, Long Evans, and Wistar rats—that may be advantageous in studying individual differences in cognitive aging trajectories. In particular, studies of outbred strains can provide insight into the genetic contributions to age-related cognitive decline and neurodegenerative disease. Regardless of wh, ether inbred, outbred, or hybrid strains are employed, researchers need to protect against gen, etic shifts or drift, which could impact reproducibility including using the same vendor and same breeding room.

The choice of an animal model may also be influenced by the cognitive processes of interest and factors that will influence outcomes and interpretation of cognitive tests. For example, strain differences can be observed in locomotor activity, stress response, memory function, and longevity (Satinder, 1981; van der Staay and Blokland, 1996; Dhabhar et al., 1997; Wolfer and Lipp, 2000; Wyss et al., 2000; Yilmazer-Hanke, 2008; Segar et al., 2009). Also, adult hippocampal neurogenesis, which declines in an age-dependent fashion, shows strong strain differences (Kempermann et al., 1997), as do synaptic markers and physiology (Guitart et al., 1992; Novick et al., 2008; Bowden et al., 2012; Paban et al., 2013; Huang et al., 2016) and morphological parameters of neurons/brains themselves (Boss et al., 1987; Wells et al., 2010).

## When Does Cognition Decline?

Relative to cross-sectional research, longitudinal studies can better identify the onset of cognitive impairment and provide a potential time course for changes in theoretical constructs of cognition and health, such as episodic memory, physical function, and circadian dysregulation. Also, longitudinal studies permit the early identification of outliers or the exclusion of adult animals that are unable to perform the task. Central for studies of brain maintenance and brain reserve, is the idea that early life events may influence the onset of age-related cognitive decline (Jagust, 2016; Hohman and Kaczorowski, 2020). Therefore, it is important to consider the phases of life in which a behavior is measured or experimental manipulation applied (e.g., Asiminas et al., 2019; Howard and Hunter, 2019). What is considered young or old may vary by animal model and strain. Rats and mice are generally weaned at ~21–28 days postnatal and reach sexual maturity by ~9 weeks of age. However, the brain is still growing at this time, and animals are not socially mature. Because many physiological, hormonal, and cognitive changes related to aging begin to occur in middle-age, longitudinal studies interested in capturing the onset of cognitive aging may want to sample more than once during middle-age. Humans undergo menopause in middle-age (~51 years). Rats and mice undergo estropause starting with irregular estrous cycles at 10–12 months, moving to an acyclic state with constant estrus (9–15 months), and follicle depletion from 18 to 24 months (Lu et al., 1979; Finch, 2014). Again, the characteristics of estropause will be strain-dependent. Finally, the definition of “old” as defined by mean/median lifespan will vary by individual strain and housing condition (Lipman, 1997; Yuan et al., 2009).

## Physical Measures, Psychological Measures, and Circadian Function

### Physical Measures

Epidemiological studies suggest measures with no clear connection to cognition can predict aging outcomes (e.g., walking speed and cognitive decline). Thus, measures with no obvious underlying construct relationship (e.g., home cage activity at middle-age and memory in old age) could prove to have substantial predictive value.

#### Body Weight

In general, in laboratory rodents’ body weight increases with age, in a sex-dependent manner (Turturro et al., 1999; Altun et al., 2007; Freund et al., 2013; Febo et al., 2020). An increase in weight could influence locomotor activity and maybe a consideration of tasks that depend on food deprivation as a motivation. Also, changes in body fat impact metabolic states. Some rat strains gain significantly more weight across age than others, such as Sprague–Dawley and Long Evans rats. One of the reasons NIA chose F344 rats for their first supported rat breeding colony is because they did not gain extreme amounts of weight across age, and thus might be preferable for some behavioral studies.

#### Grip Strength

Longitudinal studies indicate that grip strength decreases throughout aging in mice (Fischer et al., 2015; Zhang et al., 2015; Martinez de Toda et al., 2018; Sheth et al., 2018), rats (Xue et al., 2016; Febo et al., 2020) and humans (Zammit et al., 2019). In humans, grip strength measured late in life is predictive of cognitive decline (Praetorius Bjork et al., 2016; Stijntjes et al., 2017; McGrath et al., 2019). In animal models, there is little indication that changes in grip strength during middle-age predict the trajectory of cognitive decline (O’Connell et al., 2019; Febo et al., 2020).

#### Rotarod and Gait Analysis

An age-related decline in motor coordination leads to problems in the ability to maintain a uniform gait and balance during walking. Studies in humans demonstrate greater cognitive performance decrements under dual-task conditions that involve combined cognitive and motor performance (Seidler et al., 2010). Rodents exhibit age-related changes in gait (Zhang et al., 2015; Bair et al., 2019) and performance on the rotarod (Fischer et al., 2015; Zhang et al., 2015; Febo et al., 2020); however, it is not clear from longitudinal studies that motor coordination performance predicts cognitive function.

#### Locomotor Activity

In general there is a decrease in locomotor activity with age (Salvatore et al., 2009; Stowie and Glass, 2015; Febo et al., 2020). Repeated testing over the lifespan does not appear to influence sensory-motor function, such that the decline in locomotor activity is similar for longitudinal and cross-sectional studies (Altun et al., 2007). Longitudinal studies indicate that decreased locomotor activity can be observed during exploration of a novel environment (Dellu et al., 1994; Dellu-Hagedorn et al., 2004; Altun et al., 2007; Chiquita et al., 2019; Febo et al., 2020) or in an activity wheel (Dawson and Crowne, 1988; Febo et al., 2020). Continuous access to a running wheel, however, partly prevented the age-related decline in adult hippocampal neurogenesis, suggesting sustained effects of physical activity on age-dependent changes in plasticity (Kronenberg et al., 2006).

It has otherwise been suggested that physical activity and cognitive function decline in parallel in aging rodents (Shoji et al., 2016; Logan et al., 2018; Febo et al., 2020). Age-related cognitive decline was evident across multiple tasks and cognitive domains, including spatial memory performance on the Barnes maze, watermaze, and a contextual fear memory test, and executive function on reward-based discrimination and reversal learning task, which corresponded to a decrease in general activity metrics (i.e., distance traveled, acceleration). Moreover, female rodents are more active than males (Stowie and Glass, 2015; Rosenfeld, 2017; Febo et al., 2020) and sex differences in cognition may be confounded by the differential influence of exercise (Barha et al., 2017). Also, the level of estradiol is linked to activity and cognitive function. Estradiol treatment of older animals promotes wheel-running activity (Stern and Murphy, 1972; Gerall et al., 1973; Gentry and Wade, 1976) and memory (Yonker et al., 2006; Bean et al., 2015). In a longitudinal study, it was observed that activity and spatial memory function on the delayed-matching-to-place watermaze in females declined in parallel, from 12 to 18 months (Febo et al., 2020). The results suggest that longitudinal studies should examine ovarian estradiol levels as a mechanism determining sex differences in activity and memory function during aging.

#### Visual Acuity

Aged animals are more likely to develop cataracts. Albino animals may exhibit decreased visual acuity; however, effects of aging on visual acuity and memory can be distinguished by careful behavioral testing, such that impaired learning and memory are not due to the decline in visual acuity (Lindner and Gribkoff, 1991; Weber et al., 2015). A longitudinal study indicated no loss of visual acuity, as measured by the animal’s ability to perform on the visual discrimination version of the watermaze (Markowska and Savonenko, 2002). Also, age-related hearing loss has relevance for certain learning and memory tasks as was alluded to earlier (C57BL/J6 hearing loss), which is relevant to humans because hearing loss is a risk factor for cognitive decline.

#### Sleep

Sleep duration decreases with age in humans and rodent models, and the pattern of sleep (sleep stage bouts and duration) is altered such that sleep fragmentation increases (Zepelin et al., 1972; Rosenberg et al., 1979; Van Gool and Mirmiran, 1983). A longitudinal study in mice confirmed increased sleep fragmentation with age and indicated an age by sex difference for total sleep time (Fischer et al., 2015). The decrease in bout duration within different sleep stages is associated with impaired retention of inhibitory avoidance, and impaired spatial learning (Markowska et al., 1989; Stone et al., 1989). Sleep patterns are influenced by stress and environmental enrichment; however, the malleability of sleep architecture may change with age (Mirmiran et al., 1982; Buechel et al., 2014). A decrease in sensitivity to stress-induced sleep disruption may be an early marker of aging (Hargis et al., 2018). Interestingly, variability in memory in middle-age predicted changes in paradoxical sleep and sleep/circadian adaptation with advancing age (Stone et al., 1997; Febo et al., 2020).

### Psychological and Affective Measures (Response to Novelty, Anxiety, Neophobia)

#### Open Field

Locomotor activity in response to a novel environment and the time spent in the center of the open field has been used as measures of anxiety and neophobia. Individual differences in response to a novel environment, measured in adults, predict the propensity for drug abuse and memory function (Dellu et al., 1994; Antoniou et al., 2008; Flagel et al., 2014; Febo et al., 2020). Cross-sectional studies indicate that impaired learning of the reference memory version of the watermaze in older animals is associated with decreased activity in the open field, possibly due to heightened neophobia (Gallagher and Burwell, 1989; Rowe et al., 1998; Collier et al., 2004). For studies that involve multiple age cohorts, decreased exploratory activity observed in such tests may be related to a general decrease in locomotor activity characteristic of aging. Also, the oldest animals may experience prolonged exposure to an impoverished environment, which can increase neophobia/anxiety (Hall et al., 1997; Hellemans et al., 2004) and impair cognition (Diamond, 1990; Winocur, 1998; Bell et al., 2009; Diniz et al., 2010; Volkers and Scherder, 2011; Sampedro-Piquero et al., 2014; Sparling et al., 2018; Wang et al., 2018).

Cross-sectional studies examining age differences in exploration on the open field task indicate that exploration is decreased, increased, or not changed with age (Miyagawa et al., 1998; Boguszewski and Zagrodzka, 2002; Torras-Garcia et al., 2005; Bergado et al., 2011; Meyza et al., 2011; Moretti et al., 2011; Shoji and Miyakawa, 2019). Longitudinal studies in mice and rats indicate a decline in distance and velocity in the open field consistent with a general decline in activity with age (Chiquita et al., 2019; Febo et al., 2020). However, it is also possible that the decrease in locomotor activity may represent decreased anxiety due to repeated testing/handling and environmental enrichment. Environmental enrichment is associated with decreased anxiety (Fox et al., 2006), and the effects on anxiety can be observed when environmental enrichment is initiated in older animals (Galani et al., 2007; Leal-Galicia et al., 2008; Hughes and Collins, 2010; Sampedro-Piquero et al., 2014). Similarly, familiarization with the testing procedure or an age-related decrease in activity was thought to underlie an age-related decrease in arm entry for a longitudinal study of plus-maze behavior (Andrade et al., 2003).

Longitudinal studies indicate an age-related decrease in general activity (e.g., wheel running) and decreased locomotion in response to a novel environment does not correlate with impaired memory; rather, increased activity measured in adults was associated with poorer memory when tested in older animals (Dellu et al., 1994; Febo et al., 2020). It has been suggested that the increase in locomotor activity, initially seen in adults, may be due to a baseline difference in spatial learning ability, resulting in poorer habituation to exploration, which manifests as a cognitive impairment during aging. Also, the novelty induced increase in locomotor activity in a subset of adults referred to as high responders is associated with an elevated stress response highlighted in a longitudinal brain imaging study, which found that high responders to chronic unpredictable stress paradigm exhibited increased functional connectivity and atrophy within networks involved in learning and memory (Magalhaes et al., 2018). Together, the results highlight differences in the response to novelty for cross-sectional and longitudinal studies. Moreover, for longitudinal studies, individual differences in reactivity to novelty or stress in adults may be related to maintenance or reserve mechanisms, which predict network changes associated with a decline in cognitive function.

#### Visual Platform Training on the Watermaze

The initial exposure to the watermaze represents a novel environment, which is stressful to many animals (Harrison et al., 2009). Cross-sectional studies indicate task order effects mediate age-related differences when visual discrimination on the watermaze is examined before hidden platform testing. The age difference may be due to differences in anxiety in response to the watermaze, failing to shift from an inefficient strategy (i.e., thigmotaxis; Treit and Fundytus, 1988; Herrero et al., 2006). If visual platform training is initiated after hidden platform training, age differences are minimized (Rapp et al., 1987; Guidi et al., 2015). Similarly, if spatial discrimination testing is conducted after visual discrimination training, age-differences in spatial learning are reduced (Guidi and Foster, 2012). The results indicate that prior experience with the watermaze facilitates subsequent performance, possibly due to the acquisition of the procedural aspects of the task, which minimizes age differences. Thus, a longitudinal study found no age-related decline in visual discrimination performance when animals were repeatedly tested on the visual discrimination version of the watermaze (Markowska and Savonenko, 2002).

## Cognitive Measures: Hippocampus-Dependent Spatial Memory

Spatial reference and episodic/working memory depend on the hippocampus. Cross-sectional studies indicate that both decline over the course of aging; however, the training procedures and age at which impairment occurs are different for these two behaviors (Foster, 2012b). In most cases, tasks for spatial reference memory examine trial-independent and incremental acquisition over days of training (i.e., rate of learning) for invariant spatial information. In contrast, tasks for spatial episodic/working memory focus on trial-dependent and delay-dependent memory for rapidly acquired and flexible spatial information. Impaired episodic/working memory emerges earlier, possibly in middle-age, and before impairment in the acquisition of spatial reference memory. The difference in onset may arise due to the level of cognitive processing or difficulty of the tasks, or differential aging of the mechanisms involved in each process (Foster, 2012b). Alternatively, the decline may be progressive, such that impairment in rapid episodic memory may advance to more severe deficits, observed as an impaired ability to acquire spatial information through incremental learning. In this case, impairment in spatial episodic/working memory may precede and predict the trajectory of impaired spatial reference memory.

Important for longitudinal studies, tasks that involve the repeated acquisition of rapidly acquired and flexible spatial information exhibit minimal carryover effects. Also, longitudinal studies of spatial episodic/working memory, examined on several tasks (Y-maze, non-matching in a T-maze, radial arm maze, and episodic/working memory versions of the watermaze), confirm that memory deficits emerge around middle-age and continue to decline with advanced age (Ando and Ohashi, 1991; Vallee et al., 1999; Markowska and Savonenko, 2002; Dellu-Hagedorn et al., 2004; Sabolek et al., 2004; Febo et al., 2020).

In contrast to tasks that focus on delay-dependent memory, longitudinal studies emphasize that learning is resistant to age-related impairment when the procedures or strategies for solving spatial tasks are acquired in youth. These savings are particularly evident for the acquisition of spatial reference memory. Thus, relative to when rodents were first tested as young adults, aged animals exhibit no deficit and, in many cases, aged animals are better able to perform a spatial reference memory task when examined on the T-maze (Ando and Ohashi, 1991; Dellu et al., 1997), radial arm maze (Beatty et al., 1985; Bierley et al., 1986; Caprioli et al., 1991) and watermaze (Algeri et al., 1991; Pitsikas et al., 1991; Gyger et al., 1992; Dellu et al., 1997; Vallee et al., 1999; Markowska and Savonenko, 2002; van Groen et al., 2002; Vicens et al., 2002; Birch and Kelly, 2019).

### T-Maze

Several longitudinal studies have employed various types of the T-maze to examine cognition (Ando and Ohashi, 1991; Dellu et al., 1997; Zhuo et al., 2007; Talboom et al., 2014; Birch and Kelly, 2019). Ando and Ohashi (1991) first employed the delayed non-matching to place the T-maze task to longitudinally examine spatial working and reference memory. In this case, the stem of the T-maze was divided into two paths and the end of one path was always blocked, such that the animal had to acquire a trial-independent memory (i.e., reference memory) for accessing the choice point. For the trial-dependent memory, the animal (female F344 rat) was forced to select one arm, which contained a reward. After a delay, the animal was allowed to choose between the two arms, and choosing the opposite arm was rewarded. The results indicate that spatial episodic/working memory, but not reference memory declined with age. Importantly, the authors found the same result for a cross-sectional study and noted that more severe food deprivation, from 80 to 70% of free-feeding body weight, was required to get the same rate of learning in older rats (Ando and Ohashi, 1991).

Another study employed 2 days of training in which the rats learned to choose one rewarded arm of the T-maze. On the third day, the goal was shifted to the opposite arm (Dellu et al., 1997). The number of trials to reach the criteria on initial learning and reversal were tested at 3, 12, and 27 months. Acquisition improved with age (i.e., fewer errors with age) suggesting that animals remembered the procedural aspects of the task. In contrast, age-related impairments were observed for reversal learning. Similarly, training on a reference memory version of the T-maze at 6 months improved reference memory performance examined at 18 months, relative to rats initially trained on the task at 18 months (Talboom et al., 2014). Interestingly, the researchers found evidence that, for females, but not males, the cognitive training or increased handling associated with prior testing protected against age-related decline examined on other mazes. Finally, environmental enrichment was associated with protection against an age-related impairment in working memory, tested on the T-maze (Birch and Kelly, 2019).

Together, the results indicate that, for longitudinal studies, the T-maze is sensitive to impairment in episodic/working memory, confirming impaired working memory deficits observed in cross-sectional studies. The episodic/working memory deficits were observed in the absence of impairment in reference memory. The lack of impairment for reference memory is in contrast to cross-sectional studies and suggests that differences in the acquisition of a reference memory may relate to long-term memories for the procedural aspects of the task. Interestingly, environmental enrichment prevented impaired episodic/working memory, suggesting possible brain maintenance or cognitive reserve mechanisms. Important caveats for future studies include age-related changes in motivation due to food restriction and possible sex differences. Also, it would be interesting to determine if impairment in spatial episodic/working memory that emerges in middle-age could predict later deficits in spatial learning (i.e., reference memory) examined using a different task.

### Radial Arm Maze

For this task, animals are food-deprived and trained to visit equidistantly spaced arms, which radiate from a central platform, to obtain food. Performance is defined by the ability to remember which arms normally contain food and which arms have already been visited during the ag trial. In the case of the 8-arm radial arm maze, early (2–6 months) and continuous training across the lifespan can provide skills or strategies that promote learning, enabling animals to more rapidly reacquire the task later in life. Also, the use of a behavioral strategy may mask impaired working memory (Beatty et al., 1985; Bierley et al., 1986; Caprioli et al., 1991). The development of a learning strategy can be minimized by decreasing the training trials (e.g., one trial/day for 9 days) and intermittent testing (e.g., once every 6 months). In this case, the number of errors across 9 days of training increased with age such that 26-month rats made more errors than when they were tested at 3 months (Dellu et al., 1997). In another study, both working memory and reference memory improved from adult to middle-age, and decline from middle-age to old age (Templer et al., 2019). To prevent the use of response strategies, which could compensate for a decline in working memory, it is suggested that researchers increase the number of arms from 8 to 12 and control the initial arm selections (Chrobak et al., 1995; Sabolek et al., 2004). Under these conditions, older animals exhibit impairments for information acquired during the initial pre-delay training trial relative to their performance as adults.

### Y-Maze

In general, the Y-maze provides measures of spatial episodic/working memory. An important consideration is a delay between acquisition and retention testing. In one longitudinal study in mice, short-term working memory, examined as the number of alterations within a session (i.e., spontaneous alternation), was not altered with age (Chaney et al., 2018); although, there may be strain differences (Neuner et al., 2019b). In rats, variability in alternation behavior in middle-age predicted future memory deficits for tasks that impose longer delays (Stone et al., 1997). Another version of the Y-maze permits animals to explore two arms. After a delay, animals are allowed access to all three arms, and the percentage of visits and time spent in the novel arm is measured. In this case, recognition of the novel location is defined as novel arm visits greater than chance (i.e., >33%), and longitudinal studies indicate that memory declines with age (Dellu et al., 1994; Vallee et al., 1999). Interestingly, Dellu et al. (1994) found an age-related difference in memory was predicted by the locomotor response to novelty measured as adults. The memory decline was evident in older high responders following a 4 h delay, but not after a 1 min delay, suggesting that the deficit was due to impaired delay-dependent memory and not due to the impaired acquisition.

### Watermaze

A great wealth of information related to hippocampus-dependent memory has been generated in rodent models using the spatial watermaze, first developed by Morris (1984). The proper procedures for employing the watermaze when examining aged animals have been detailed elsewhere (Foster, 2012b; Guidi and Foster, 2012; Guidi et al., 2014; Burke and Foster, 2019). Therefore, the current review focuses on the use of the watermaze for longitudinal studies. Cross-sectional studies indicate that deficits in the acquisition of a spatial reference memory can be observed in a subset of older animals (Gallagher et al., 2015). Other studies suggest that with advanced age, the majority of the oldest animals exhibit impairments in forming a reference memory, depending on the task parameters (Lindner and Gribkoff, 1991; Clark et al., 1992; Rick et al., 1996). In contrast, longitudinal studies emphasize that when the procedures for spatial reference memory are acquired in youth, performance is resistant to age-related impairment, such that aged animals are better able to perform a spatial reference memory task on the watermaze relative to when they were first tested as young adults (Algeri et al., 1991; Pitsikas et al., 1991; Gyger et al., 1992; Dellu et al., 1997; Vallee et al., 1999; Markowska and Savonenko, 2002; van Groen et al., 2002; Vicens et al., 2002). In mice from 6 to 14 months, working memory on the T-maze declined in the absence of impaired reference memory on the watermaze (Zhuo et al., 2007), confirming the vulnerability of working memory. Finally, while repeated reference memory training across the lifespan was protective against learning deficits in older rats, performance at 12 months could predict performance at 18 months (Markowska and Savonenko, 2002). Similarly, aged animals (24 months) that were impaired in the reference memory version of the task exhibited savings in learning when retested in a new environment 2 weeks later; however, a probe trial 30 min after the final training trial indicated that those aged rats exhibiting the most impairment during the initial training also were the most impaired during transfer training (Colombo and Gallagher, 2002).

There is evidence that aging mice and transgenic Alzheimer’s disease (AD) mice may not exhibit the same carryover/protective effects on learning when testing is initiated in adults (Vicens et al., 2002; Billings et al., 2005; Reichel et al., 2017). One study in particular investigated the relationship of hippocampal volume and an age-related impairment of spatial reference memory (Reichel et al., 2017). A retrospective examination of mice that exhibited poor learning at 24 months indicated these same animals exhibited poorer learning at 16, but not 8 months. Interestingly, no difference in hippocampal volume was observed at 16 months; however, poor performers exhibited a greater decrease in the volume of the dorsal hippocampus from 16 to 24 months. The results suggest that the emergence of cognitive deficits may signal underlying processes that ultimately result in a decline in hippocampal volume.

Episodic/working memory can be examined using the delayed-matching-to-place (DMTP) version of the watermaze or a 1-day version of the watermaze. For this task, the platform is moved for each session. A session consists of an acquisition phase, followed by a retention test after a delay. Longitudinal studies confirm that memory deficits begin to emerge in middle-age, particularly for longer delays, and the propensity for memory deficits increased with age, such that more aged animals exhibit impairments at shorter delays (Febo et al., 2020). The deficits in middle-age correlated with time spent in the center of the open field. Interestingly, deficits were more apparent for males and correlated with their response to a novel environment measured at 6 months. In the case of females, memory function in middle-age predicted disturbances in circadian adaptability with advanced age.

For the 1-day version of the watermaze, training occurs in a single day. An acquisition probe trial can be delivered after several blocks of training to determine the learning; a second retention probe trial can be delivered after a delay (2–24 h) to measure memory. The task can be repeated, with the escape platform located in a different position during retesting. The task is reliable for measures of memory, such that behavior on the retention probe trials were correlated when the task was repeated with a 10-day interval (Guidi et al., 2014). Another study found that performance on the acquisition probe trial at 12 months could predict probe trial performance at 18 months (Markowska and Savonenko, 2002). It is important to note that acquisition and retention probe trials should be followed by a refresher block of training with the return of the escape platform, to ensure that the animals do not shift their search strategy. Also, there may be a limit on the number of probe trials that can be administered. At some point, the animals may figure out that sometimes, the platform goes missing and turns up in another location.

### Barnes Maze

The Barnes maze is a dry-land test of spatial memory, in which the animals need to navigate a circular surface with holes around its circumference to find an escape tunnel to leave a brightly lit, exposed surface (Barnes, 1979). Thus, the animal must remember the escape tunnel location, using spatial cues in the room. This maze is less stressful than the watermaze (Harrison et al., 2009). However, similar to training on the watermaze, early training on the Barnes maze results in better learning later in life due to preserved procedural memory (Heimer-McGinn et al., 2020). For aged animals, the Barnes maze often is the method of choice, if the watermaze cannot be applied because of its greater physical demands (Berdugo-Vega et al., 2020).

### Pattern Separation

An age-related decline in the ability to distinguish objects as feature overlap increases or discriminate between the locations of two adjacent identical stimuli often referred to as pattern separation has been well documented in both humans (Yassa and Stark, 2011; Holden and Gilbert, 2012) and animal models (Gracian et al., 2013; Johnson et al., 2017; Ces et al., 2018; Smith et al., 2020). Experimental evidence supports a role for the dentate gyrus and CA3 subregions of the hippocampus in pattern separation (Wilson et al., 2006; Creer et al., 2010; Wu et al., 2015; Ces et al., 2018; Ray et al., 2018; Smith et al., 2020). Additionally, the dentate exhibits early markers of aging including altered synaptic plasticity and a decline in neurogenesis. Currently, we are unaware of any longitudinal studies to determine the onset and trajectory of pattern separation impairment in an animal model.

## Prefrontal Cortex Based Tasks

The prefrontal cortex mediates executive functions, a comprehensive term used to describe several cognitive abilities required to accomplish goal-directed behavior (Bizon et al., 2012; Burke and Foster, 2019). The set of cognitive processes include sustained attention, working memory, behavioral inhibition, impulse control, and cognitive flexibility. In the case of aging, deficits in basic cognitive processes (e.g., processing speed or attention) may emerge early and contribute to impairments in executive processes, while other basic cognitive components, such as signal detection or maintenance of information in short-term memory remain intact (Goh et al., 2012; McAvinue et al., 2012). In humans and animal models, age-related deficits in prefrontal tasks are observed as attentional demand is increased.

Sustained attention involves the maintenance of vigilance over time. Older humans exhibit little or no decrement for tasks that require the selection of relevant stimuli (Glisky, 2007). However, impairments are observed for tasks that require dividing or switching of attention among multiple inputs as conditions are changing. In this case, improvement in sustained attention is observed during the maturation of adults, into their 30 s, and deficits begin to appear starting in middle-age (McAvinue et al., 2012; Fortenbaugh et al., 2015). Similarly, longitudinal studies indicate that aging rodents can maintain selective attention on some tasks (Burk et al., 2002).

Age-related and pathology related deficits are reported for the 5-choice serial reaction time task (5-CSRTT). For this task, attention is focused on lighted ports such that attention is spatially divided among five different locations. The animal must respond to the light (nose poke) to obtain a reward (Burke and Foster, 2019) For this task, vigilance is measured as responses (accuracy, response latency, omissions) to multiple cue/response ports, and attentional demand is increased by shortening the cue duration (0.5–0.25 s). The 5-CSRTT has been employed to longitudinally track vigilance in AD mouse models (Lambourne et al., 2007; Bharmal et al., 2015) and a Huntington’s disease mouse model (Yhnell et al., 2016). Also, longitudinal 5-CSRTT studies in rats have examined an age-related decline in vigilance (Muir et al., 1999). Similar to cross-sectional studies, longitudinal studies indicate that impairment emerges in middle-age when the attentional load is increased by decreasing the cue duration (Muir et al., 1999). For a cue of 0.5 s the young and aged animal’s performance was ~≥70% correct. Both groups exhibited a decrease in performance as the duration decreased to 0.25 s with greater impairment in older animals. Impairment was not due to sensory-motor function as response latency was not different and performance was not affected by changing the cue brightness. In contrast, another longitudinal study found no decline in vigilance during aging (Grilly et al., 2000). In this case, rats were initially trained at 10–11 months and retrained every 8–10 months until they were 34–35 months. Several important differences may explain the absence of an age-related impairment. First, animals were maintained on food restriction for the duration of the study; although, the role of caloric restriction in cognitive decline is debatable (Ingram and de Cabo, 2017). More importantly, the cue duration (>1 s on average) was adjusted to maintain correct responses within 75–88%. Compared to animals that initially learned the task (10–11 months) or were retrained in middle-age (18–19 months), older animals (24–25 and 34–35 months) appear to require twice as many training sessions to reach criteria. Thus, aged animals were able to reach the criteria by adjusting for a bias against slower learning in older animals. Finally, the use of a longer duration cue may have reduced the attentional demand to within the threshold for aged animals. As noted above, age-related impairments are observed as the attentional demand is increased by decreasing the cue duration to below 1 s. Together, the results support the idea that age-related impairment in vigilance can be observed as attentional demand is increased (i.e., reducing the cue duration).

Cognitive flexibility, examined by the attentional set-shifting task, refers to the ability to alter behavior in response to changes in goals or environmental cues, permitting the rapid adaptation of behavior to a change in contingencies. For example, the ability to shift from responding with a nose poke to a port signaled by light cue, regardless of port position (left or right), to respond to a rewarded position (right port) regardless of the presence or absence of the previously rewarded light cue (Burke and Foster, 2019). Attentional s, et-shifting was shown to decline with age (6–14 months) in a longitudinal study in mice (Zhuo et al., 2007). Also, these same animals exhibited spatial working memory deficits on the T-maze, but no spatial reference memory impairment, examined on the watermaze.

Working memory involves several component processes including encoding, maintenance of information for short intervals (seconds), and the manipulation of information. For aging humans, deficits are minimal or non-existent for passive maintenance of information (e.g., forward digit span) and robust deficits are observed for the task that examines the capacity of working memory, consistent with the idea that deficits are observed as attentional demand is increased (Bopp and Verhaeghen, 2005; Glisky, 2007; Alexander et al., 2012; Brockmole and Logie, 2013; McNab et al., 2015). Due to difficulties in isolating the prefrontal cortex and hippocampal contributions to spatial working memory examined on the T-maze, Y-maze, radial arm maze, and DMTP tasks, operant-based paradigms have been employed to focus on visual discrimination and minimize spatial discrimination (Burke and Foster, 2019).

### Delayed Response

For animal models, maintenance of information in working memory is examined using delayed-response tasks. In this case, mnemonic demand is increased by increasing the delay between the sample and choice trials. In a delayed non-matching to position task, F344 × BN rats were tested each month from 28 to 34 months (Blokland et al., 2004). This task involved an operant box with two levers. During the sample phase, one lever was presented and a response (lever press) resulted in a reward. After a delay, both levers were presented during the choice phase and a response to the lever in the sample phase resulted in a reward. A clear decrease in correct responses was observed as the delay increased; however, performance did not decline over the 6 months of testing.

### Span Task

Examination of working memory capacity provides another method for increasing mnemonic demand. Working memory capacity is examined using span tasks in which the animal must remember a series of stimuli; mnemonic demand is taxed by increasing the number of memoranda to be encoded into and maintained by working memory stores. For non-spatial tasks, working memory capacity, rather than the length of time that information can be maintained in memory is important in contributing to general cognitive function (Kolata et al., 2005, 2007). In this case, animals respond to a stimulus (e.g., digging in a scented bowl). With each subsequent trial, another novel stimulus is added to the array of previous stimuli. The novel stimulus is the only one rewarded for a response. The trials continue until the animal makes an error, responding to a previous stimulus. The number of stimuli remembered (i.e., before an error) provides a measure of working memory capacity (Dudchenko et al., 2000). Few studies in rodents have examined age-related changes in the capacity of working memory, with the possible exception of spatial working memory on the radial arm maze. As noted above, increasing the number of arms from 8 to 12 may increase the demand for spatial working memory (Sabolek et al., 2004). Longitudinal studies in monkeys indicate that the decline in working memory capacity, is highly variable and in some cases, the influence of practice effects cannot be ruled out (Moss, 1993; Koo et al., 2018; Ibanez et al., 2019).

### Delay Discounting

Delay discounting is a measure of impulsivity and cross-sectional studies indicate impairment with age (Simon et al., 2010; Hernandez et al., 2017). This task usually involves an operant chamber. A signal indicates that the two response levers or ports are active. A response one port (immediate) results in an immediate, yet small reward. In contrast, a delayed response (e.g., 5 s to the port designated as the delayed port results in a much larger reward. We are unaware of longitudinal animal studies examining delay discounting; however, it is important to note considerable genetic differences in the ability of rats and mice to tolerate a delay (Isles et al., 2004; Anderson and Woolverton, 2005; Wilhelm and Mitchell, 2009). Importantly, delay discounting distinguishes impulsive choices from impulsive actions. While enhanced ability to delay gratification is not in and of itself evidence of behavioral impairment, at least one cross-sectional study (Hernandez et al., 2017) revealed that enhanced delay discounting among aged rats is also associated with maintained working memory, diminished cognitive flexibility, and greater break-points (or willingness to work for food rewards) on a progressive-ratio task. The relationship of delay discounting with the maintenance or decline in other prefrontal-mediated cognitive processes, suggests that longitudinal studies of delay discounting may provide an opportunity to predict the onset and trajectory of cognitive decline in specific prefrontal functions.

## Perirhinal Cortex Based Tasks

### Novel Object Recognition

The novel object recognition task (NOR) depends on an animal’s propensity to explore novel objects and is used as a measure of recognition memory. The task consists of two phases, a familiarization phase in which the animal is allowed to explore duplicate copies of an object (e.g., miniature figure). The animal is then removed from the apparatus. Following a delay (30 s to 24 h) the animal is returned to the apparatus for the test phase, in which one of the objects has been replaced with a novel object (Burke and Foster, 2019). Depending on the task parameters, NOR performance may differentially involve the perirhinal cortex, prefrontal cortex, and/or hippocampus (Hammond et al., 2004; Albasser et al., 2009, 2015; Barker and Warburton, 2011; Cohen et al., 2013). A longitudinal study found that environmental enrichment improved performance on the NOR task and prevented an age-related decline in object recognition (Birch and Kelly, 2019). Several longitudinal studies indicate that previous training on the task can influence performance throughout aging. In a mouse study, NOR with a 1 h delay was examined at 6, 12, and 18 months (Chaney et al., 2018). For wild-type mice, memory performance appeared to increase from 6 to 12 months indicating a savings effect due to previous training. However, the performance was not different from the chance at 18 months, indicating impaired recognition, despite no age-related difference in time exploring the objects. Similarly, other longitudinal studies find improvements due to previous training (Chiquita et al., 2019; Marshall et al., 2019). Thus, previous experience influences performance possibly due to positive transfer.

## Other Considerations

### Food Restriction

For some tasks, food restriction is employed to motivate animals to perform. Food restriction or intermittent feeding raises a potential confound because the caloric restriction is known to influence the rate of aging, decreasing inflammation, and improving healthspan and can modify brain markers of aging (Fontana et al., 2018; Mattson et al., 2018). Several studies have examined longitudinal food restriction on learning and memory in animal models of aging. In general, improvement is observed for motor function; however, the effect of food restriction on cognitive function, including learning is unclear, with little or no benefit observed for memory, and in some cases impairment (Ingram and de Cabo, 2017). Thus, there appears to be an interesting and important disconnect between the effects of lifelong caloric restriction on health and biological markers of aging and cognitive decline. Other studies suggest that di, et influences brain aging and cognitive relationships in middle-age (Adams et al., 2008; Granholm et al., 2008; Li et al., 2013; Yegla and Foster, 2019) and any benefit may decline in the oldest animals (Stewart et al., 1989). Finally, the effects of food restriction on biomarkers and behavior are confounded by an increase in general motor activity, as the foraging behavior of hungry animals is converted into increased locomotor activity (Cui et al., 2009). Besides, food restriction can also modify locomotor activity on tasks that involve spontaneous exploration (Y-maze, novel object recognition). In this case, animals may be motivated to explore/forage due to their fasted status (Carter et al., 2009).

Important for longitudinal studies is research that employed food restriction as a motivation for spatial episodic/working memory (Ando and Ohashi, 1991; Chrobak et al., 1995; Dellu et al., 1997; Sabolek et al., 2004; Templer et al., 2019), vigilance (Muir et al., 1999; Lambourne et al., 2007; Bharmal et al., 2015; Yhnell et al., 2016), and set-shifting (Zhuo et al., 2007), which continue to find age-related cognitive decline. Furthermore, it is possible that once the animals acquire the task in their youth, reinstatement of behavior in middle-age animals will be facilitated, reducing the time of food restriction required to accomplish the testing. Thus, it may be possible to have short periods of food restriction only during times of testing. In this case, researchers may want to also include a task that declines with age but does not require food deprivation (e.g., Y-maze, DMTP on the watermaze) to determine if food restriction has a general effect on the rate of brain aging and cognitive decline.

### Health Status

The trajectory of cognitive decline depends on health status (Anstey and Christensen, 2000; Stewart et al., 2000; Wahlin et al., 2006) and undetected health problems may add to variability in behavior (Spangler and Ingram, 1996; Febo et al., 2020). Cardiovascular disease (Bink et al., 2013), kidney disease (Vlassara et al., 2009), and metabolic diseases, diabetes and insulin resistance (Biessels and Gispen, 2005; Greenwood and Winocur, 2005; Mattson and Arumugam, 2018), as well as the history of systemic inflammation, can have a negative impact; accelerating cognitive decline. However, examining the relationship between cognition and mortality may be difficult as the identification of a moribund state may be problematic if animals do not show obvious clinical signs of disease (Snyder et al., 2016). Interestingly, a cross-sectional study examining behavior/cognition concerning pathology indicated that morbidity determined by gross necropsy does not contribute to cognitive decline (Spangler et al., 1994).

### Order of Behavioral Tests

For studies that involve multiple behavioral tests, the order of behavioral testing may result in qualitatively and quantitatively different outcomes. For the watermaze, aged animals may exhibit a poor initial strategy and delayed acquisition of the procedural aspects of the task. Thus, initial testing of visual vs. spatial discrimination can facilitate subsequent performance on the other task, minimizing age differences in visual discrimination (Rapp et al., 1987; Guidi et al., 2015) or spatial discrimination (Guidi and Foster, 2012). Also, some tests are sensitive to stress, such that disruption may occur if a stress-sensitive test is preceded by a test that involves considerable stress. One recommendation is to order the test from least stressful to most stressful. For example, initially testing spontaneous behavior, Y-maze, NOR, or the open field, followed by more stressful tests (e.g., watermaze visual discrimination followed by spatial discrimination) and behaviors that require food restriction. Also, the number and duration of tests will determine how often testing can be repeated. This is a likely consideration for tasks that involve food restriction and extensive behavioral shaping.

### Behavior as a Predictor of Brain Maintenance and Cognitive Reserve

An important question for longitudinal studies is whether physiological, psychological/affective, or early behavioral/cognitive measures can predict the onset and trajectory of cognitive decline. Early predictors may reflect innate differences that promote brain maintenance or brain reserve mechanisms. For example, performance on the reference memory task at 12 months predicted performance at 18 months (Markowska and Savonenko, 2002), suggesting the presence of pre-existing, baseline differences as animals matured from adulthood to early middle-age. A baseline difference, before brain aging or pathology, which results from genetics, sex, or differential experiences in youth that modifies the brain and associated cognitive processes, would suggest the involvement of a brain or biological reserve mechanism. As such, this brain/biological reserve mechanism may delay the onset of brain aging and cognitive decline. Indeed, differences in genetic makeup are likely to play a large role in maintaining the brain and determining susceptibility to age-related cognitive decline and neurodegenerative diseases of aging (Neuner et al., 2019c; Dunn et al., 2020).

Related to the question of whether behavior can predict cognitive decline and associated maintenance or reserve mechanisms, is the idea that different cognitive functions or networks might decline in parallel or that different strategies involving different brain circuits may be employed to compensate for cognitive/network deficits. Many age-related changes are not independent of each other. The underlying direct or shared causality that is implicated by observed correlations is difficult to untangle. Here, data from longitudinal animal cohort studies combined with advanced multivariate and non-linear modeling (e.g., structure equation modeling, SEM) will allow progress, as has been the case in human studies, but with much greater control of independent variables and experimental parameters.

Several longitudinal studies found that impulsivity or an increased locomotor response to novelty, measured in young adult rats, predicted poorer memory during aging (Dellu et al., 1994; Dellu-Hagedorn et al., 2004; Febo et al., 2020). Also, the performance of young animals on the 1-day version of the watermaze (Hullinger and Burger, 2015) or rate of learning on a T-maze (Talboom et al., 2014) predicted better performance at older ages, and better spontaneous alternation in middle-age predicted better inhibitory avoidance memory with advanced age (Stone et al., 1997). In many cases, the tasks involve delay-dependent memory, suggesting that the predictive behavior and impaired cognitive function depend on the same network or process. In contrast, a decline in working memory on the T-maze (Muir et al., 1999; Zhuo et al., 2007) and impaired vigilance on the 5-CSRTT did not predict reference memory deficits on the watermaze, suggesting dissimilarities in cognitive processes and brain networks or differences in cognitive demand across tasks. Additional predictive factors and their combinations likely exist and multivariate cohort studies would have the power to identify these factors.

Existing studies tend to restrict the range for possible predictors to the variable immediately at hand. However, some tasks have substantial carryover effects, such that these tasks may not be suitable for repeated testing in longitudinal studies. As noted above, different conclusions may be drawn among cross-sectional and longitudinal studies that examine the reference memory version of the watermaze, the radial arm maze, or NOR performance. One possibility may be to use different tasks that theoretically measure the same cognitive process at different time points. For example, acquisition of a spatial reference memory can be measured on the watermaze and the Barnes maze, and both tasks exhibit carryover effects associated with the acquisition of procedures for spatial reference memory. However, cross-sectional studies indicate little predictability between tasks (Gallagher and Burwell, 1989; Markowska et al., 1989; Arendash and King, 2002; Leighty et al., 2004). In contrast, aged female C57BL/6J mice exhibited impairment for the watermaze and poorer retention of inhibitory avoidance (Benice et al., 2006). However, others report no relationship between learning on the watermaze and inhibitory avoidance in aging rats (Markowska et al., 1989; Blokland and Raaijmakers, 1993). The results emphasize that individual differences do not always correlate across tasks due to dissimilarities in cognitive demand or the sensitivity of each task for a specific cognitive process (Foster, 2012b).

Better correspondence may be observed when both tasks focus on delay-dependent retention or forgetting. It should be noted that for these tasks, cognitive demand (i.e., the duration of the delay) can be manipulated to equate difficulty across tasks and maximize individual variability. Aged (26 months) Wistar rats exhibited deficits for retention of inhibitory avoidance and retention of a spatial reference memory when retention was examined 6 days following the end of watermaze training (Miettinen et al., 1993). Similarly, retention of spatial memory on the 1-day version of the watermaze task correlated with retention of inhibitory avoidance in aged (18–23 month) F344 rats (Foster and Kumar, 2007). In a study of middle-aged (12 months) Long-Evans female rats, poor retention was observed for the watermaze, inhibitory avoidance, and object recognition (Paris et al., 2011). Finally, for middle-age (14 months) and aged (24 months) male F344 rats, impaired retention 24 h following a single day of watermaze training was related to the 24 h retention performance on a novel object recognition task (Blalock et al., 2003). The correspondences across multiple tasks that measure the same theoretical construct provide some validity for the idea that the tasks involve the same neural network or process.

### Treatments or Interventions to Influence Brain Maintenance and Cognitive Reserve

In addition to relevant variables (age, sex, and strain) and measures of brain aging or pathology (see below), and cognitive function (physical, psychological and cognitive), longitudinal studies are likely to address the role of independent variables and positive or negative modifiers, which could underlie the individual differences in age-related cognitive decline and resilience or plasticity mechanisms that preserve cognitive function. An important aspect of longitudinal studies will be the determination of when treatments are viable and likely to be most effective in modifying aging trajectories. Treatments delivered during developmentally sensitive periods may influence the behavior of adults and have long-term positive effects to maintain the brain and cognition or negative effects that increase susceptibility to aging and pathology. Lifelong environmental enrichment was associated with preserved recognition memory, spatial working memory, and longer-term (24 h) spatial memory (Birch and Kelly, 2019). Longitudinal training, starting at 3 months, on tasks that tax prefrontal function through increased attentional demand and working memory, was associated with preserved working memory and learning with advancing age (Matzel et al., 2011).

Furthermore, plastic processes triggered by aging or pathology may shift with advancing age such that beneficial treatments delivered in youth or adulthood may not induce resilient, adaptive, or compensatory cognitive reserve mechanisms in aged animals. Several studies have reported that late-life environmental enrichment improved learning and memory, suggesting that cognitive reserve mechanisms that are sensitive to the environment are intact with age (Kumar et al., 2012; Speisman et al., 2013; Sampedro-Piquero et al., 2014, 2015; Neidl et al., 2016; Cortese et al., 2018; Balietti et al., 2019). It should be noted that in most cases, the environmental enrichment also decreased anxiety suggesting a link between psychological and cognitive measures. However, one study examined middle-aged (17 months) female rats, previously characterized as impaired or unimpaired on the reference memory version of the watermaze. In this case, environmental enrichment for 6 months preserved long-term memory (10 days post-training) examined at 24 months only in animals characterized as unimpaired at 17 months (Fuchs et al., 2016), suggesting an inability for environmental enrichment to access cognitive reserve mechanisms in animals that already exhibit cognitive decline on this task.

## Assessment of Biomarkers of Aging

In addition to characterizing cognitive variability in terms of the trajectory of cognitive performance, and determining when treatments are effective in promoting cognitive reserve, it will be important to define markers of biological aging, particularly within brain networks for cognitive processes of interest. Biomarkers can be employed to understand individual variability in the onset and trajectory of brain/cognitive aging and to differentiate normal aging from diseases that affect cognition. The development of sound biomarkers of biological or functional aging is required to determine whether preserved cognition for a specific chronological age is due to brain maintenance (i.e., an absence or delay in brain aging) or plastic changes that preserve cognition in the face of brain aging (i.e., cognitive reserve). For longitudinal studies, minimally invasive measures are preferable, since these measures are nominally disruptive to the study and can be translated across species. An understanding of the relationship between cognition and biomarkers of aging/cognition that are translatable across species could be employed to determine who might be helped from a specific intervention. Minimally invasive measures include neural imaging and biomarkers found in blood samples; although, other measures (e.g., microbiome) are being developed. The development of valid noninvasive biomarkers will permit the examination of cellular, molecular, and epigenetic mechanisms of brain aging and cognitive reserve. Thus, correlations between cognition and neuroimaging during aging could be employed to determine when and where to examine cellular and molecular changes. This paradigm could bridge a localized cellular/molecular phenomenon with systems neuroimaging and cognition measures.

### Brain Imaging

Among the various noninvasive techniques, magnetic resonance imaging (MRI) and magnetic resonance spectroscopy (MRS) of the brain, are uniquely suited for longitudinal measurements; providing in-depth assessments of neural activity, relatively high resolution of tissue microstructural organization and chemistry linked to neurons, glia, and oxidative stress in the aging brain (Febo and Foster, 2016). Thus, an understanding of the time course for changes in cognition and in functional connectivity or brain structures can provide information concerning possible cognitive reserve mechanisms, and determine when and where to examine molecular mechanisms. Structural imaging studies indicate that whole-brain volume reaches maximal levels by about 14 months in mice (Maheswaran et al., 2009) and rats (Sullivan et al., 2006). Longitudinal studies of aging and mouse models of neurodegenerative disease can track changes in brain metabolism, and brain volume and the effect of interventions (Holmes et al., 2016; Peng et al., 2018; Birch and Kelly, 2019). In an AD mouse model, an early (4 months) decrease in hippocampal volume was associated with impaired NOR performance (Chiquita et al., 2019). In contrast, a longitudinal study of normal aging indicated that memory impairment observed in middle-aged mice predicted accelerated dorsal hippocampal volume loss in older animals (Reichel et al., 2017). In this case, it is likely that senescent physiology underlies cognitive impairment and contributes to later volume loss.

Animal imaging approaches combining functional MRI (fMRI) with behavioral characterization may be used to investigate the relationship between regional changes in senescent physiology (i.e., neural activity) and structural connectivity, bringing us closer to localizing changes in neural circuits that underlie cognitive impairment or that represent compensation and altered efficiency to adjust to pathology/aging within brain networks. In rodents, studies of functional connectivity have focused on the resting state due to technical limitations in examining behaving animals. Altered resting-state functional connectivity between the hippocampus and several other brain regions, including increased connectivity with the retrosplenial cortex (RSC), was associated with the emergence of impaired watermaze DMTP performance in middle-age (Febo et al., 2020). In humans, an increase in functional connectivity, which is observed early in association with memory impairment, may predict further cognitive decline, reduced functional connectivity, and loss of brain volume with more advanced age (Reuter-Lorenz and Cappell, 2008; Wang et al., 2011; Staffaroni et al., 2018; Zheng et al., 2018). Similarly, in a rat model of AD, higher dorsal hippocampal connectivity in middle-age progressed to reduced dorsal hippocampus-RSC connectivity with age (Parent et al., 2017) and older rats characterized as impaired on a spatial reference memory task exhibited decreased dorsal hippocampus-RSC connectivity (Ash et al., 2016). The latter study also reported altered functional connectivity specifically associated with preserved memory in aged rats. Finally, it will be important to determine whether the relationship between functional connectivity, loss of volume, and cognitive decline are linked to other relevant variables that might influence the preservation of cognition including anxiety and the response to stress or novelty (Magalhaes et al., 2018, 2019).

While rodents can be acclimated to the fMRI recording procedures, to provide recordings from awake animals (King et al., 2005; Zhang et al., 2010; Becerra et al., 2011; Upadhyay et al., 2011; Jonckers et al., 2014), most rodent studies use anesthesia protocols to minimize movement and physiological variations across subjects during scanning (Liu et al., 2013; Jonckers et al., 2014; Ash et al., 2016; Febo and Foster, 2016; Huang et al., 2016; Parent et al., 2017; Magalhaes et al., 2019). In this case, it is important to consider the level of anesthesia during scanning when interpreting fMRI results in rodents. For resting-state functional connectivity, isoflurane is employed and the level is below that which reduces the sensitivity of spontaneous BOLD activity and the use of seed-based correlations to reveal functionally connected networks similar to that observed in humans. These parallels between human and rodent resting-state networks hold promise for the use of functional connectivity to track age-associated cognitive network changes. Finally, it should be acknowledged that there may be the potential concerns of repeated anesthesia effects, in addition to anesthesia × age interactions.

### Blood Biomarkers

Currently, there is considerable effort to identify blood biomarkers to estimate biological age, track the progression of cognitive decline, and monitor the effectiveness of treatments. Repeated measures of blood-based biomarkers in rodents may be limited due to the amount of blood required/available. Previous longitudinal studies in rodents have mainly focused on markers of longevity (Acuna-Castillo et al., 2006; Ho et al., 2019). Thus, a blood-based biomarker concerning cognitive function represents an important area for exploration in future longitudinal studies.

While the exact nature and mechanisms for aging continue to be debated, important markers of biological age might include blood measures of genomic instability, epigenetics, and cellular senescence (Lidzbarsky et al., 2018; Niedernhofer et al., 2018; Aristizabal et al., 2020), telomere length (Vera et al., 2013; Stauffer et al., 2018), inflammation and the proinflammatory secr, etory profile designated the senescence-associated secretory phenotype (SASP; Boersma et al., 1985; Heller et al., 1998; Guayerbas et al., 2002; Moeller et al., 2014; Martinez de Toda et al., 2016), hormone levels (Gee et al., 1983; Matt et al., 1986; Smith et al., 1992; Fentie et al., 2004), mitochondrial function, redox state, and antioxidant defense (Heller et al., 1998; Stauffer et al., 2018; Jain et al., 2019). For example, levels of corticosterone in the blood have been associated with memory function during aging (McQuail et al., 2018; Huzard et al., 2020).

Brain-specific proteins and microRNAs (miRNAs) found in the circulation can act as biomarkers for brain injury and diseases of aging. In blood, brain proteins and miRNA are found packaged into small (50 nm to 1 μm) extracellular vesicles that are released from cells into the extracellular environment. Some (e.g., exosomes) can cross cell membranes and deliver their contents to influence cell health (Barter and Foster, 2018; White et al., 2019). Intercellular communication from tissue to tissue by exosomes is a potential mechanism that would connect peripheral activity, exercise, and systemic inflammation, to overall brain health. In turn, exosomes produced by and released from the brain may represent biomarkers of brain function.

### Cellular and Molecular Markers

Due to the invasive nature of studies that examine cellular and molecular mechanisms, cross-sectional studies in inbred or isogenic lines, involving the same environmental background, are considered pseudo-longitudinal and have been used to examine cellular and molecular mechanisms associated with age and cognitive decline. In some cases, subsets of animals, selected at specific ages can be used for terminal sampling of molecular and cellular measures in the brain and blood (Achin et al., 2018). In the case of brain function, studies have focused on senescent physiology involving a shift in synaptic plasticity and altered cellular and neuronal network activity, dysregulation of Ca^2+^ homeostasis, oxidative stress, inflammation, and neurogenesis (Gray and Barnes, 2015; Haberman et al., 2017; Mattson and Arumugam, 2018; Dunn and Kaczorowski, 2019; Foster, 2019; Oh and Disterhoft, 2020). As discussed below, the relationship between cellular and molecular markers of aging and cognitive decline may define aspects of brain maintenance and cognitive reserve.

## Mechanisms for the Preservation of Cognitive Function: Brain Maintenance and Cognitive Reserve in Animal Models

There are at least two ways to preserve cognitive function during aging: brain maintenance and cognitive reserve and these processes are not mutually exclusive (Table 2). Brain maintenance relates to factors (genes, sex, early life intervention, or differential experiences), that delay or prevents both cognitive decline and brain changes associated with aging and pathology. In contrast, cognitive reserve preserves cognition by initiating plastic or compensatory processes, in many cases, triggered by aging or pathology. Thus, for brain maintenance, larger differences in biological measures are observed between young and aged-impaired, with minimal differences in biological measures of aging between young and aged-unimpaired. In contrast, cognitive reserve may involve a response to aging such that larger differences in biological measures are revealed between young and aged-unimpaired. Implicit in the definitions is the idea that brain maintenance occurs before aging or pathology to delay aging, while the cognitive reserve is activated during aging as a plastic response to the stressors of aging or pathology. However, brain maintenance and cognitive reserve likely involve similar underlying mechanisms. For example, treatments delivered during aging that can act to rejuvenate the brain and rescue cognition would promote brain maintenance. Similarly, cellular resilience, a protective cellular response triggered by aging or pathology, while considered a reserve mechanism, could delay markers of brain aging. Thus, similar life exposures can impact different measures of brain maintenance and mechanisms of cognitive reserve; however, the relationship between cognitive reserve and maintenance will depend on the measures employed. Finally, it is likely that the plasticity mechanisms of cognitive reserve decline with advancing age or become overwhelmed by aging and pathology.

**Table 2:** Strategies of preservation of cognitive function.

Domain	Feature	Characteristics related to the preservation of cognitive function
Brain maintenance (slowing/prevention of decline and pathology)
	Resting-state functional connectivity	Maintained CA3 > CA1 connectivity (Liang et al., 2020).
	Gene expression changes	Maintenance of immune, synaptic, cholesterol/lipid metabolism pathways (Burger, 2010; Smith et al., 2020).
Cognitive reserve (adaptation/plasticity to maintain cognitive function in the presence of aging and pathology)		
	Cellular resilience	Regulation of Ca^2+^ homeostasis, inflammation, oxidative stress, cellular waste disposal pathways (Blalock et al., 2003; Neuner et al., 2016, 2019a,c; Mattson and Arumugam, 2018; Foster, 2019; Smith et al., 2020).
	Network efficiency	Increased efficiency (i.e., less activation) in CA1, mPFC (Kelly and Deadwyler, 2002; Paban et al., 2013; Sampedro-Piquero et al., 2015; Tomas Pereira et al., 2015; Ianov et al., 2016; Yagi et al., 2016; Hernandez et al., 2018; Smith et al., 2020).
	Network capacity	Increased capacity (greater activation) in the dentate gyrus during spatial memory tasks (Yau et al., 1996; Vann et al., 2000; He et al., 2002; Blalock et al., 2003; Colombo et al., 2003; Palop et al., 2003; Small et al., 2004; Rowe et al., 2007; Deipolyi et al., 2008; VanElzakker et al., 2008; Penner et al., 2011; Marrone et al., 2012; Gheidi et al., 2013; Fletcher et al., 2014; Benito et al., 2015; Weber et al., 2015; Bernstein et al., 2019).
	Network compensation/flexibility	Shift to non-hippocampal memory systems to compl, ete task (allocentric > egocentric, CA1 > basal ganglia, amygdala activation; Cabeza et al., 2002; Burger et al., 2007; Deipolyi et al., 2008; Grady, 2008; Epp and Galea, 2009; Steffener et al., 2009; Morris et al., 2012; Olvera-Cortés et al., 2012; Steffener and Stern, 2012; Zelikowsky et al., 2013; Snigdha et al., 2017; Cabeza et al., 2018; Pignataro et al., 2019; Smith et al., 2020).

### Brain Maintenance

Brain maintenance relates to factors (genes, sex, early life intervention, or differential experiences), that slow or prevent both cognitive decline and brain changes associated with aging and pathology. The emphasis lies on change over time. Thus, brain maintenance may be defined as the preservation of cognitive function associated with minimal changes in brain markers of aging or pathology. In this case, aged-impaired animals are expected to exhibit biological measures of the effects of aging or pathology that are different from young animals. However, these age differences are not as marked between young and aged unimpaired animals. Thus, brain maintenance represents an absence of or resistance to develop age-related brain changes or pathology. Perfect brain maintenance would result in no cognitive decline and no age-related brain changes. In practice, this is observed as a decrease in the rate of brain aging (Rapp et al., 2020). For example, preserved learning associated with maintenance of resting-state functional connectivity (Liang et al., 2020), In this case, aged impaired animals exhibited reduced hippocampal CA3 connectivity with region CA1 and its connectivity with the infralimbic prefrontal cortex compared to young animals. In aged animals with preserved learning, connectivity was more akin to that of young animals. Similarly, gene expression studies have identified region-specific age changes (i.e., aging genes), in which the largest changes are observed for animals that are impaired on tasks that depend on the specific region examined (Burger, 2010; Smith et al., 2020). In many cases, the expression is related to specific biological processes that change with age. Animals with preserved cognition appear to have better maintenance of gene expression within molecular pathways that mediate the inflammation/immune response, maintain synaptic structure and function, cholesterol/lipid metabolism and preserve neurogenesis. In addition to changes associated with aging, variability in biological measures may be secondary to cognitive differences. Thus, aged animals that are unable to form new memories are likely to exhibit differences in measures of synaptic structure, signaling (e.g., CREB phosphorylation), and gene transcription normally induced by learning.

Several cross-sectional studies have examined behavioral/cognitive, dietary, and pharmacological interventions to test the idea that treatments that preserve cognition are associated with minimal age-related changes in neural circuits, gene expression, and physiological function, particularly synaptic plasticity. For example, synaptic plasticity correlates with cognitive function in models of aging, stress, and neurodegenerative disease. The weight of evidence supports the idea that interventions associated with better cognitive function are also associated with the maintenance of synaptic plasticity. A handful of longitudinal studies, testing the same animals at multiple ages, indicate beneficial effects on cognition observed for a hypocaloric diet (Algeri et al., 1991) and social housing (Templer et al., 2019). However, these longitudinal studies did not examine biological markers of brain aging. Environmental enrichment and cognitive stimulation can induce clear differences in cognitive function, in some cases reversing age-related cognitive deficits (Milgram et al., 2006; de Villers-Sidani et al., 2010; Kumar et al., 2012; Birch and Kelly, 2019; Gelfo, 2019; Kempermann, 2019), improving disease-related phenotypes (Wood et al., 2011; Wassouf and Schulze-Hentrich, 2019) and reversing some aging markers (Kumar et al., 2012; Speisman et al., 2013; Park et al., 2015; Stein et al., 2016; Birch and Kelly, 2019). Lifelong environmental enrichment and increased locomotor activity are associated with an increase in neurogenesis that normally declines with age (Freund et al., 2013; Birch and Kelly, 2019). Nevertheless, it is unclear which aspects of environmental enrichment; social interaction, cognitive stimulation, exposure to novelty, and increased motor activity, contribute to improved cognitive function (Kumar et al., 2012). Also, it is not clear what cognitive process could be influenced by changes in neurogenesis during aging (Bizon and Gallagher, 2005; Martinez-Canabal et al., 2013; Speisman et al., 2013; McGuiness et al., 2017; Ray et al., 2018; Toda and Gage, 2018; Smith et al., 2020). Moreover, how these manipulations work to counteract the mechanisms of aging and pathology may remain to be determined; although, it is likely that these factors act on aging processes, decreasing oxidative stress and inflammation, and improving metabolism (Foster, 2006).

Brain reserve may represent a subcategory of brain maintenance in that the genetic, or treatment factors that delay cognitive and brain changes also influence cognition in adults. Thus, for brain reserve, better cognition is associated with baseline differences in adults. In turn, behavioral differences in adults may predict the trajectory of cognitive decline (Dellu et al., 1994; Markowska and Savonenko, 2002; Dellu-Hagedorn et al., 2004; Talboom et al., 2014; Hullinger and Burger, 2015; Febo et al., 2020).

### Cognitive Reserve

The research aimed at further elucidating cognitive reserve requires the inclusion of three components: the status of the brain (reflecting age-related brain change or pathology), clinical or cognitive performance changes/outcomes, and a measure of reserve. The cognitive reserve represents plasticity properties of the brain that allow for sustained cognitive performance in the face of age-related brain changes or pathology that would normally be associated with cognitive impairment. Here the emphasis is on potential, not on actual change over time. Thus, processes related to cognitive reserve are operationally identified as measures that explain individual variability in cognition that are not due to chronological age or pathology alone. Ideally, the aim is to demonstrate that the proposed cognitive reserve measure moderates the relationship between an indicator of brain aging, abnormality, or pathology and clinical/cognitive status. That is, cognitive performance should be predicted by the interaction between that proposed factor and brain/pathology status. It can also be sufficient to demonstrate that a hypothesized cognitive reserve measure is associated with cognitive performance after parceling out the effects of brain change, pathology, or insult. For example, in human studies, a multiple regression analysis predicting cognition that includes brain atrophy/pathology measures and a hypothesized cognitive reserve proxy, the proxy should account for additional predictive variance (Steffener and Stern, 2012; Hohman et al., 2016). In this analysis, the new cognitive reserve proxy adds predictive information (a protective factor). Below we discuss possible cognitive reserve mechanisms at different levels of biology. However, in each case, the cognitive reserve mechanism represents a form of plasticity that is initiated by aging processes and helps to preserve cognitive function, which might otherwise be impaired by aging or pathology. The plasticity can occur at the molecular/cellular level in response to molecular mechanisms of aging, structural/functional adaptation at the circuit level, or the recruitment of brain networks, not normally employed to perform a task, to compensate for the disruption of the network that normally underlies task performance.

### Cellular Resilience in Response to Aging

Cellular resilience is triggered early in the course of aging or pathology and represents attempts to mitigate the molecular processes of brain aging: mitochondrial dysfunction, oxidative stress, the need for increased cellular waste disposal, Ca^2+^ dysregulation, and inflammation (Mattson and Arumugam, 2018). As such, measures of cellular resilience associated with preserved cognition may signify cognitive reserve. In contrast to brain maintenance, in which there is little difference in biological measures between young and cognitively unimpaired, cellular resilience is likely to involve differences that distinguish cognitively unimpaired animals from young, as well as cognitively impaired aged animals that are not able to mount a cellular response. Transcriptional profiling early in aging or early in the progression of neurodegenerative disease, suggests that preserved cognition involves transcription of genes that regulate biological processes of aging including the regulation of Ca^2+^ homeostasis and cell excitability, regulation of inflammation or cellular damage, oxidoreductase enzymes, and specific organelles for waste disposal (lysosome, exosome, mitochondria; Blalock et al., 2003; Neuner et al., 2016, 2019a,c; Foster, 2019; Smith et al., 2020). However, with more advanced age or increasing pathology, cellular resilience mechanisms may fail to preserve cognition (Hargis and Blalock, 2017; Foster, 2019).

Treatments that tap into cellular resilience to preserve cognition could include hormesis, an adaptive response to a low-level stressor, which protects against the stressors of aging or pathology (Calabrese, 2013; Brose et al., 2018). For example, mild metabolic or oxidative stress can trigger the induction of genes that increase stress resistance and improve cognition (Brose et al., 2018; Mattson and Arumugam, 2018). Exercise or environmental enrichment is associated with transcriptional changes related to processes that change with age (Tong et al., 2001; Park et al., 2015; Grinan-Ferre et al., 2016; Huttenrauch et al., 2016; Berchtold et al., 2019). Again, the effectiveness of some treatments may decline with advanced age, suggesting a loss of biological plasticity (Kuhla et al., 2013). Agents that mimic the effects of exercise or caloric restriction, in addition to providing a potential treatment, could provide means for measuring cellular resilience and predicting the cognitive trajectory.

### Local Circuit/Network Implementation of Cognitive Reserve

In human studies, a goal of longitudinal studies has been the identification of functional networks or circuits, either resting or task-related, whose expression moderates the relationship of brain senescence or pathology to cognition. In considering these mechanisms, a distinction might be made between the adaptation of the typically used local circuits and the recruitment of additional networks. For circuit adaptation, the same circuit is employed by young and aged animals; however, differences in the efficiency or capacity of the circuit underlies the variability in cognition. In contrast, network compensation involves the use of alternative networks, and strategies to compensate for deficits in the circuit normally employed for the task.

### Circuit/Network Adaptation

The idea that cognitive reserve may be based on the differential efficiency/capacity of a circuit is largely based on neuroimaging in humans, examining brain activation as cognitive demand is varied (Stern, 2009). Efficiency/capacity is defined by the level of neural activity in a brain region that underlies the cognitive process of interest. Young individuals likely can modulate the efficiency/capacity of a circuit and this plastic property is diminished with age or pathology. Testing these ideas in animals with parallel methods is limited by the fact that behaving animals are not readily amenable to neuroimaging (Febo and Foster, 2016). However, animal models permit the examination of cellular and molecular markers of neural activity. Thus, neural activity related to task performance can be measured using electrodes, localized to specific synapses and circuits to measure local field potential, electroencephalographic recordings, and the discharge activity of neurons in behaving animals. Molecular signatures of neural activity include expression of activity-dependent immediate-early genes (*Fos*, *Arc*, *Zif-268*).

*Efficiency* can be defined as the degree to which a given task-related brain circuit must become activated to accomplish said task. A more efficient network will show less activation to produce the same (or better) level of performance. Thus, an individual with greater efficiency will show less task-related neural activity at a given level of task demand. Interestingly, for animal studies that involve extensive exposure to the same environment, use of an allocentric spatial strategy, superior spatial memory, and preserved cognitive flexibility are associated with decreased *Fos* (Yagi et al., 2016; Smith et al., 2020) and *Arc* expression (Kelly and Deadwyler, 2002; Tomas Pereira et al., 2015) in region CA1 of the hippocampus, suggesting that prior experience increased efficiency of the system, facilitating spatial memory. Within the medial prefrontal cortex of young rodents, enhanced performance on a radial arm watermaze, following environmental enrichment, was associated with decreased *Fos* expression in the infralimbic region of the prefrontal cortex (Sampedro-Piquero et al., 2015), again suggesting the possibility that training or experience can increase the efficiency of the circuit, observed as a lower activity linked to better performance. One prediction might be that variability in the cognitive function of older animals will be linked to the ability to harness this form of plasticity. Interestingly, in aged animals, increased expression of immediate early genes in the medial prefrontal cortex is associated with decreased cognitive flexibility, impaired working memory, and a slower rate of learning (Paban et al., 2013; Tomas Pereira et al., 2015; Ianov et al., 2016; Hernandez et al., 2018). The results are consistent with the idea that the impairment is due to an inability to access plastic properties for increased efficiency within a circuit that underlies the behavior.

*Capacity* can be defined as the maximum degree to which a task-related brain network can be activated to perform a task in the face of increasing demands. For several tasks described above, altering cognitive/attentional demand can reveal variability in age-related cognitive impairment; however, the link between task demand and biological parameters is rarely measured in animals. In contrast, several studies have examined immediate-early genes to determine the difference in baseline activity or the level of activity induced by learning in impaired and unimpaired animals. Expression of the activity marker *Fos*, either the mRNA or the protein, in various hippocampal regions is increased as animals learn about novel environments and novel objects (He et al., 2002; VanElzakker et al., 2008; Bernstein et al., 2019) and as the requirement for spatial memory is increased (Vann et al., 2000; Colombo et al., 2003). In AD mouse models that exhibit impaired use of a spatial strategy, basal levels of *Fos* are decreased in the dentate gyrus (Palop et al., 2003; Deipolyi et al., 2008). Similarly, basal *Fos* levels and induction of *Fos* by a novel environment was decreased in the dentate gyrus with age (Weber et al., 2015). Likewise, for other immediate early genes, early growth response gene, *Zif-*268 (Yau et al., 1996; Blalock et al., 2003; Rowe et al., 2007; Gheidi et al., 2013; Benito et al., 2015) and the activity-related immediate-early gene *Arc* (Blalock et al., 2003; Small et al., 2004; Rowe et al., 2007; Penner et al., 2011; Marrone et al., 2012; Fletcher et al., 2014), induction following exposure to a novel environment, as well as training-related expression are decreased in aged or age-memory impaired animals. The results suggest that cognitive reserve can be associated with mechanisms that regulate the ability to activate the system.

Local circuit adaptation can be observed at other levels of analysis and the response will depend on the stimulus (Gray and Barnes, 2015; Rapp et al., 2020). For example, aging and AD are associated with synaptic loss and local circuits may invoke plastic processes such as increasing the strength of remaining synapses (Barnes and McNaughton, 1980; Foster et al., 1991; Scheff et al., 1996; Chen and Hillman, 1999; Burke and Barnes, 2006; Neuman et al., 2015). Behavioral discrepancies are linked to plastic properties that depend on learning-induced and activity-dependent signaling cascades (i.e., CREB phosphorylation) to influence cell excitability (Dunn and Kaczorowski, 2019; Foster, 2019; Montaron et al., 2020; Oh and Disterhoft, 2020). Task-specific changes in local amygdala electroencephalogram recordings, selectively within the beta range (15–30 Hz), developed with training, selectively in aged rats, possibly as an adaptive mechanism to preserve learning (Samson et al., 2017). Also, differences in resting-state connectivity between impaired and unimpaired animals could represent a local response as an adaptation to altered input or compensation due to the recruitment of different networks (Small et al., 2004; Ash et al., 2016; Febo et al., 2020).

### Network Compensation

While cellular resilience or local adaptation in the face of age or pathological stressors can be described for tissues other than the brain, network compensation is a particularly brain-centric cognitive reserve mechanism. In response to the local disruption of a neural circuit due to aging or pathology, individuals may recruit other brain structures or networks (and thus cognitive strategies) not normally used by individuals with “intact” brains (Steffener and Stern, 2012). Compensation can result in improved performance (Cabeza et al., 2002; Grady, 2008). Alternately it could result in maintenance of performance, but perhaps at a lower level than when compensation is not required (Steffener et al., 2009). Given a specific level of aging or brain pathology; several possibilities underlie network compensation as a cognitive reserve mechanism. The greater cognitive reserve could be associated with the lack of compensation (while compensation is seen at lower levels of cognitive reserve). Alternately individuals with a higher cognitive reserve may compensate more successfully to maintain function, albeit at a lower level. The advantage of nonhuman studies is that these functional underpinnings to performance can be more directly evaluated and understood.

For animal models, compensation is well characterized for hippocampal impairments, which result in a shift in strategy and reliance on neural circuits within other memory systems. A decline in allocentric memory, linked to brain aging in region CA1 and the dentate gyrus of the hippocampus, results in a greater reliance on egocentric memory involving the basal ganglia or prefrontal cortex (Burger et al., 2007; Deipolyi et al., 2008; Olvera-Cortés et al., 2012). Similarly, an impairment in the ability to perform a pattern separation task due to damage to the dentate gyrus, decreased neurogenesis, or aging of the dentate gyrus can be compensated for, at least in part, by the use of allocentric or egocentric strategies that depend on other brain regions, including other subregions of the hippocampus (Epp and Galea, 2009; Morris et al., 2012; Snigdha et al., 2017; Smith et al., 2020). Similar to human studies, the use of compensatory cognitive processes or strategies is usually less than optimal relative to those employed by younger or intact individuals (Cabeza et al., 2018).

Several recent animal studies provide descriptions of plastic properties involved in the recruitment of different circuits to compensate for brain aging and pathology of the hippocampus (Zelikowsky et al., 2013; Pignataro et al., 2019). Contextual fear conditioning normally depends on the hippocampus and in an AD model, preserved memory was observed despite a decrease in hippocampal activation (Pignataro et al., 2019). Rather, increased activation and dendritic spine growth were observed in the amygdala. These results provide an example of how dysfunction of one element of the neural network may be compensated by recruitment of other elements to preserve memory performance. In general, the mechanism for compensation involves a shift in brain activity between different networks.

## Conclusions

Differences in the onset and trajectory of age-related cognitive decline result from factors related to the characteristics of the individual (genetics, sex, and epigenetic dispositions), the environment, and lifestyle factors, as well as behavioral activity, which interact to influence the brain. It is hoped that an understanding of how these many factors interact across the lifespan will enable interventions to maintain the brain and promote the formation of cognitive reserve, a property of the brain that allows for sustained cognitive performance in the face of age-related changes, brain insult, or disease. The current review highlights relevant variables (age, sex, and strain) and appropriates dependent variables emphasizing physical, psychological, and cognitive measures that can be examined longitudinally. Also, it will be important for future studies to identify biological markers that predict the onset or trajectory of cognitive decline. Finally, we suggest ways of operationally defining measures of brain maintenance and cognitive reserve.

## Author Contributions

All authors contributed to the article and approved the submitted version.

## Conflict of Interest

The authors declare that the research was conducted in the absence of any commercial or financial relationships that could be construed as a potential conflict of interest.

## Footnotes

^1^https://reserveandresilience.com/background/
